# Transfer Learning Fusion Approaches for Colorectal Cancer Histopathological Image Analysis

**DOI:** 10.3390/jimaging11070210

**Published:** 2025-06-26

**Authors:** Houda Saif ALGhafri, Chia S. Lim

**Affiliations:** 1 Department of Information Technology, College of Computing and Information Sciences, University of Technology and Applied Sciences, Muscat 133, Oman; 2 Graduate School of Technology, Asia Pacific University of Technology and Innovation, Kuala Lumpur 57000, Malaysia; lim.chiasien@apu.edu.my

**Keywords:** colorectal cancer, decision fusion, transfer learning, spatial attention mechanisms, histopathological images

## Abstract

It is well-known that accurate classification of histopathological images is essential for effective diagnosis of colorectal cancer. Our study presents three attention-based decision fusion models that combine pre-trained CNNs (Inception V3, Xception, and MobileNet) with a spatial attention mechanism to enhance feature extraction and focus on critical image regions. A key innovation is the attention-driven fusion strategy at the decision level, where model predictions are weighted by relevance and confidence to improve classification performance. The proposed models were tested on diverse datasets, including 17,531 colorectal cancer histopathological images collected from the Royal Hospital in the Sultanate of Oman and a publicly accessible repository, to assess their generalizability. The performance results achieved high accuracy (98–100%), strong MCC and Kappa scores, and low misclassification rates, highlighting the robustness of the proposed models. These models outperformed individual transfer learning approaches (*p* = 0.009), with performance differences attributed to the characteristics of the datasets. Gradient-weighted class activation highlighted key predictive regions, enhancing interpretability. Our findings suggest that the proposed models demonstrate the potential for accurately classifying CRC images, highlighting their value for research and future exploration in diagnostic support.

## 1. Introduction

Deep learning attention in image recognition is driven by the historical need for specialized expertise in feature extraction for classification tasks [1]. A notable decrease in error rates was reported in the ImageNet Large Scale Visual Recognition Competition (ILSVRC) [2]. Convolutional neural networks (CNNs) [3] have become a staple in computer vision due to automating feature extraction through convolutional layers, simplifying the work for professionals, and enabling broad application, especially in medical fields. Popular CNN architectures such as AlexNet [2], VGGNet [4], Inception [5], Xception [6], ResNet [7], and MobileNet [8] have been widely adopted in imaging competitions.

Colorectal cancer (CRC) is associated with rising global rates of morbidity and death [9]. The growing prevalence of CRC has been linked to modern lifestyle factors such as high intake of processed foods, alcohol consumption, physical inactivity, increasing obesity, and delayed routine screenings [10,11]. According to 2023 cancer statistics, CRC is the third in cancer incidence and second in cancer-related deaths globally. The primary objectives of CRC screening are to reduce mortality by identifying the disease early and removing pre-cancerous lesions, with colonoscopy being essential for detecting and managing suspicious lesions [12]. Excessive cell growth can lead to benign tumors like polyps or adenomas in stage 0, with around 10% potentially becoming malignant [13]. These may progress to adenocarcinoma, invading deeper tissues by stage 1, reaching the serosa in stage 2, and the visceral peritoneum in stage 3. By stage 4, metastasis to lymphatic or blood vessels may occur, according to 2024 data from the National Cancer Institute. Traditional CRC diagnosis mainly involves histopathological analysis [14], utilizing biopsy samples stained with hematoxylin and eosin [15,16]. However, this process requires expert image analysis, highlighting the growing need for automated methods in CRC histology classification [17].

Pathologists microscopically examine diseased tissues in histopathology, and the 1999 introduction of whole-slide imaging enabled the digital conversion of tissue specimens into high-quality virtual slides [18]. This advancement improves accuracy and efficiency in analysis, aiding the development of automated CRC classification methods. However, a challenge in CRC image analysis is the limited availability of annotated samples, essential for training deep learning models. These models require large datasets to prevent overfitting [19]. Transfer learning addresses the limitations of small datasets by leveraging knowledge from related domains, tasks, or scenarios to improve model performance during the training and testing phases [20,21]. Additionally, incorporating attention mechanisms enables the network to emphasize the most relevant features, therapy improving performance [14]. Inspired by the human visual search process, attention mechanisms help the model identify key areas within complex images. Specifically, spatial attention dynamically assigns weights to different image regions, guiding the model to concentrate on the most informative parts. This targeted focus not only enhances feature extraction but also optimizes computational efficiency by prioritizing critical spatial regions [22]. In parallel, as a core technique in deep learning, transfer learning enables continual learning by reusing previously acquired knowledge to solve new problems more efficiently and effectively [23]. However, CNNs require substantial data, computation, and time to perform effectively, which often struggle with limited medical image datasets [24]. 

This study addresses the challenge of improving robustness, accuracy, and interpretability in CRC histopathological image classification using deep learning. We introduce Attention Decision Fusion Models (ADFMs), which integrate spatial attention mechanisms with decision-level fusion to enhance interpretability and discriminative power. Leveraging transfer learning with pre-trained models (InceptionV3, Xception, and MobileNet), ADFMs offer a tailored solution for the complexities of CRC diagnosis. Evaluations across private, public, and composite datasets confirm the models’ robustness and generalization. The primary contributions of our study are as follows: (1) The development of three hybrid models, each combining specific pairs of pre-trained CNN architectures (InceptionV3, Xception, MobileNet) to extract complementary features from CRC histopathological images. These models use spatial attention mechanisms within each CNN branch to enhance feature representation before classification. (2) A spatial attention mechanism that prioritizes regions in histopathological images improves diagnostic accuracy. (3) The design of an attention-based decision fusion mechanism that combines and adaptively weighs the predictions from these hybrid models to generate a more accurate classification. This fusion strategy leverages the strengths of multiple models to improve robustness and reduce individual model biases. (4) A method to partition curated datasets into subsets for targeted analysis of specific histopathological features to enhance the models’ applicability across diverse healthcare settings. With these contributions, the proposed models offer a promising foundation for future advancements in diagnostic support and medical image analysis.

### Related Work

Colorectal carcinoma ranks third globally among cancer-related deaths, approximately 10% of cancer incidences, and 9–10% of such deaths [25]. This morbidity has spurred research efforts to improve diagnosis by adopting CNNs [24]. Recent research has concentrated on enhancing the automatic categorization of CRC subtypes to reduce inter- and intra-variability among pathologists [26]. One challenge faced in this domain is the scarcity of large-scale datasets, which has led to an increased emphasis on publicly accessible datasets. In 2019, Ref. [27] introduced a histological dataset specifically for classifying CRC subtypes, facilitating research in automated image analysis. The study [28] emphasized that deep neural networks provide superior capacity and generalization compared to shallow networks. Their findings show CNNs outperform traditional algorithms and human capabilities when trained on large datasets.

Transfer learning, especially when combined with fine-tuning, is widely adopted in deep learning-based CRC diagnosis due to its ability to transfer knowledge from large-scale image datasets to smaller, domain-specific medical datasets [29]. For example, Ref. [30] applied transfer learning using pre-trained CNN models (VGG16, ResNet50, and adaptive ResNet152) for multiclass classification of CRC histopathology images across eight tissue types. While their method achieved high accuracy by freezing specific layers to control the flow of information, such fixed-layer strategies may limit model adaptability to complex histological variations. Similarly, Ref. [31] compared ResNet18 and ResNet50 on the Warwick-QU dataset, showing that deeper architectures provided improved performance. However, both studies primarily relied on standard pre-trained networks without significant architectural customization or validation across varied datasets.

Numerous studies have explored CNN-based architecture for CRC classification, emphasizing the potential of deep learning in histopathological image analysis. For instance, Ref. [32] proposed a 45-layer CNN for colon cancer classification, while [33] developed a framework for distinguishing cancerous tissue types, further demonstrating the effectiveness of deep models in pathology. Similarly, Ref. [34] experimented with architectures ranging from 10 to 16 layers to improve detection performance. Despite the architectural variety, many of these studies focus on single datasets or lack systematic validation across tissue subtypes, raising concerns about overfitting and limited generalizability.

Other works have relied on well-known pre-trained models. In [35], Inception V3 was used with a highly annotated proprietary dataset, while [36] evaluated five CNNs on multiple public datasets, and ResNet50 outperformed the rest. In [37], a fine-tuned VGG19 model achieved 91.2% accuracy within nine epochs by applying overfitting mitigation strategies. Likewise, Ref. [38] utilized ResNet50 to classify non-overlapping patches from whole-slide CRC images.

Ensemble models have also emerged as promising strategies. For example, Ref. [39] combined VGG19, DenseNet201, and EfficientNetB7, achieving 94.17% accuracy using a concatenated ensemble. However, ensemble methods often demand greater computational resources, posing practical challenges for clinical deployment.

Furthermore, attention mechanisms are gaining popularity for their ability to focus on salient features, mimicking human visual processing. In [24], channel and spatial attention modules were integrated into VGG16, achieving 87.3% accuracy and employing Grad-CAM for interpretability. Nonetheless, the attention maps in such studies are rarely cross-validated with expert annotations, leaving their clinical relevance uncertain.

Despite notable progress in deep learning for CRC histopathological image classification, key gaps remain in enhancing model robustness and interpretability. Notably, decision fusion strategies where outputs from multiple models are integrated to form a consensus prediction are underexplored in this domain, despite their demonstrated benefits in improving classification accuracy and reducing model bias [40,41,42]. This is particularly important in medical imaging, where inter-patient variability and histological heterogeneity can compromise the performance of single models. Additionally, while attention mechanisms have shown promise in other computer vision applications, their use in CRC diagnosis remains limited. Our work addresses these shortcomings by employing a decision fusion framework that leverages the complementary strengths of different models and integrates attention-based modules to refine the spatial focus of features. Through this dual strategy, our approach advances CRC image classification by improving robustness, interpretability, and consistency across diverse datasets.

## 2. Materials and Methods

Unlike traditional ensemble techniques, such as hard and soft voting, as well as stacking, which are limited by fixed or equal weighting schemes that fail to account for varying model reliability across inputs [43], our proposed attention-driven fusion strategy directly addresses these limitations by learning dynamic, instance-specific weights for each model’s prediction. We concatenate prediction scores and pool attention-weighted features from multiple pre-trained models (InceptionV3, Xception, MobileNet) and apply a learnable attention layer to fuse their outputs adaptively. This allows the network to emphasize stronger model predictions and downweight weaker ones depending on the input, improving decision fusion beyond conventional ensemble strategies.

**Figure 1:**
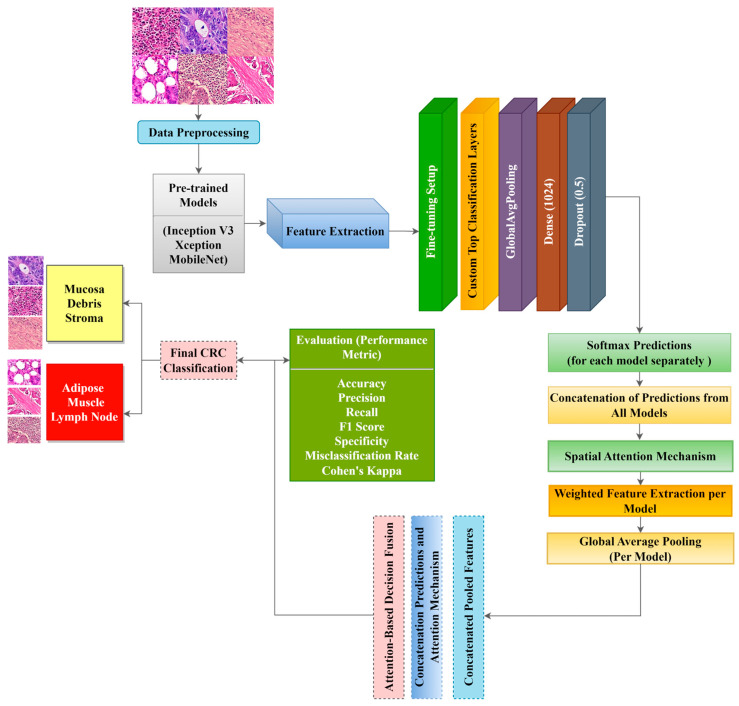
Mechanism of the ADFM approach for CRC histopathological image classification. Each model processes histopathological image patches through two specific pre-trained CNN backbones: ADFM 1 (InceptionV3–Xception), ADFM 2 (Xception–MobileNet), and ADFM 3 (InceptionV3–MobileNet). Feature maps from both branches are enhanced using spatial attention mechanisms. The fused features are passed through fully connected layers for final classification into six datasets of colorectal tissue types: mucosa, stroma, debris, lymph, muscle, and adipose. This architecture integrates multi-scale feature extraction, spatial attention, and decision fusion to improve robustness and accuracy.

We proposed three ADFMs (ADFM1, ADFM2, and ADFM3) to enhance the classification accuracy of CRC histopathological images. Each proposed model integrates different combinations of state-of-the-art models, InceptionV3, Xception, and MobileNet, to exploit feature extraction capabilities and achieve high performance. ADFM1 combines InceptionV3 and Xception, leveraging InceptionV3’s ability to capture multi-scale features and Xception’s depthwise separable convolutions for detailed spatial feature extraction. ADFM2 fuses Xception with the lightweight MobileNet, balancing computational efficiency and classification accuracy. ADFM3 integrates InceptionV3 with MobileNet, utilizing InceptionV3’s complex feature extraction alongside MobileNet’s efficiency for resource-constrained environments. For all models, the lower layers of each pre-trained network are frozen, preserving the learned feature representations from large-scale ImageNet data. In contrast, the upper layers are fine-tuned to adapt to the CRC-specific task. The transfer learning approach ensures the retention of generic visual features while tailoring the networks to the task of CRC classification. Figure 1 illustrates the architecture of all three ADFMs, showing the flow of input images through the paired pre-trained models, the application of spatial attention, and the fusion mechanism that leads to the final classification decision.

InceptionV3 and Xception are deep architectures capable of extracting high-level features. The final convolutional layers of InceptionV3 and Xception output a feature map of (5 × 5 × 2048) and (7 × 7 × 2048), respectively. MobileNet is a lightweight network with depth-wise separable convolutions, making it computationally efficient with a final feature map of (7 × 7 × 1024). For the ADFMs, we applied a spatial attention mechanism by using a Conv2D(1,1) with sigmoid activation, generating attention maps of sizes (5 × 5 × 1) and (7 × 7 × 1). These maps are then element-wise multiplied by the initial feature maps to highlight relevant regions. The refined feature maps undergo global average pooling, producing feature vectors of 1 × 2048, 1 × 2048, and 1 × 1024 from Inception V3, Xception, and MobileNet, respectively. These vectors are concatenated and passed through fully connected layers, followed by attention-weighted fusion, where learned weights would determine the contribution of each model’s prediction to the final decision. The main advantage of the approach is the use of spatial attention mechanisms, which enhance critical areas related to CRC histopathology. Additionally, the weighting mechanism allows predictions from different models to contribute dynamically based on their importance. The approach provides a robust hybrid architecture that balances accuracy, computational efficiency, and interpretability for classifying CRC histopathological images. This process is outlined in Table 1, where attention weights are applied to scale the predictions from the two models before summing them to form the final decision.

### 2.1. CNN Architectures with Transfer Learning

The CNN architecture used in this study focuses on transfer learning for classifying CRC histopathological images. While CNNs generally require large, annotated datasets, such as ImageNet [44], medical imaging datasets are often limited. Transfer learning addresses this challenge by enabling models to leverage pre-trained weights, extracting features without needing extensive domain-specific data. This approach transfers knowledge from one model to another for related tasks, enhancing performance by utilizing prior learning from large datasets [39,45,46,47,48].

This study leverages transfer learning to address limited data in CRC histopathology classification by fine-tuning pre-trained models, reducing data requirements and computational cost. We employed models including Inception V3, Xception, and MobileNet, for feature extraction, following a widely recognized strategy in transfer learning applications [29]. The pre-trained models are modified by excluding their fully connected layers set with include_top = False, leaving the feature extraction layers intact. Custom top layers for CRC classification include a global average pooling layer, a dense layer with 1024 units and ReLU activation, and a dropout layer with a 0.5 rate. Early stopping is used to monitor training to enhance model performance, and a learning rate scheduler adapts the learning rate across training epochs. This methodology consolidates predictions from multiple models, boosting the classification accuracy of CRC histopathological images.

The following offers a concise overview of the pre-trained models used in this study. In 2016, Ref. [49] introduced InceptionV3, a novel architecture that achieved impressive accuracy in the ILSVRC 2012 classification benchmark. The distinguishing feature of the Inception model lies in its parallel configuration of convolutional layers, incorporating kernel sizes of 5 × 5, 1 × 1, and 3 × 3, along with a 3 × 3 max-pooling layer. This design allows multiple convolution filters on a single input, facilitating multi-level feature extraction while mitigating computational costs. The Xception architecture, proposed by [6], referred to as “extreme inception”, introduces a key innovation by replacing the traditional inception modules with a combination of depthwise separable convolutions followed by pointwise convolutions. This design improves computational efficiency and model performance by separating spatial and channelwise feature processing. The architecture includes 36 convolutional layers, forming the core of its feature extraction capabilities. MobileNets, introduced by [8], comprise a series of streamlined models tailored for mobile and embedded vision applications. These models employ a simplified structure incorporating depthwise separable convolutions to construct lightweight deep neural networks. The MobileNet model is built upon depthwise separable convolutions, a depthwise convolution, and a 1 × 1 convolution referred to as a pointwise convolution.

### 2.2. CRC Histopathological Datasets and Preprocessing

In this study, the CRC histopathology models using public and private datasets are split by tissue types to enhance feature learning, improve accuracy, reduce bias, and support robust, generalizable classification. The first dataset was sourced from the Royal Hospital, the pathology department, and the histopathology diagnostics laboratory, and was divided into two subsets. For diagnostic analysis, the first subset includes mucosa, debris, and stroma (Dataset 1). For staging evaluations, the second subset comprised adipose, lymph nodes, and muscle (Dataset 2). The pathologists’ expertise guided this partitioning and the dataset’s alignment with clinical objectives and diagnostic workflows. This aims to identify cancerous changes, assess tumor extent and invasion, and make treatment decisions based on the CRC stage. Each histopathological sample was also converted into smaller patches to ensure the dataset’s compatibility with computational processing and deep learning models. These patches underwent annotation utilizing QuPath 0.5.0 software [50]. This process accurately represents histopathological features across various classes within the dataset. Furthermore, all patches are standardized and resized to 224 × 224 pixels to maintain uniformity in data processing.

The second dataset is employed from the publicly available CRC-VAL-HE-7K dataset [27], which mirrors the methodology used for the private dataset, with six discrete classes delineated for Datasets 3 and 4. Integrating private and public datasets in creating the third dataset highlights a strategic effort to enhance the diversity and breadth of data sources in computational pathology. These Datasets 5 and 6 afford a rich tapestry of histopathological representations by harmonizing datasets of a diverse array of tissue types and pathological manifestations.

**Figure 2:**
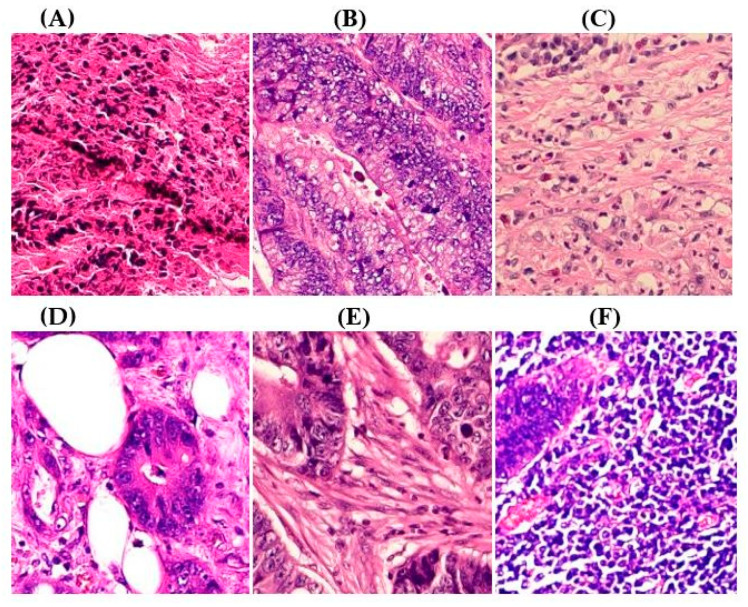
Representative samples of CRC histopathological images from the Royal Hospital. Images (**A**–**C**) depict debris, mucosa, and stroma from Dataset 1, while images (**D**–**F**) showcase adipose tissue, muscle, and lymph nodes from Dataset 2. The mucosa serves as the primary site for CRC development, while the stroma provides a structural framework that can indicates tumor invasion depth. Debris, often linked to necrotic tissue, may signify tumor progression and response to treatment. Adipose and muscle tissues are relevant in assessing deeper invasion or metastasis, whereas lymphatic tissue is essential for evaluating cancer spread to lymph nodes.

ADFM maintains preprocessing data consistency with a structured pipeline: images are loaded via OpenCV, resized to 224 × 224 × 3, and scaled to [0, 1]. Labels are encoded and one-hot transformed. The dataset is then randomly split into training (85%) and testing (15%) sets, enabling efficient and balanced analysis. We present detailed class distributions for each of the six datasets used in our study (Table 2), providing transparency regarding the representation of each class. Although formal class balancing methods were not applied, for the public dataset, class imbalance, particularly in debris and stroma in Dataset 3, was retained to preserve its original distribution. However, by using K-Fold cross-validation, we ensured that the evaluation was conducted across multiple folds, thereby reducing the effect of imbalance on model outcomes. Additionally, the patch extraction process was conducted manually by expert pathologists using QuPath, with cross-checking to ensure annotation consistency and reduce variability. While sampling biases were minimized through careful manual selection, we acknowledge that inherent dataset imbalances and potential annotation variability remain limitations and highlight these as directions for future work. Figure 2A–F present representative samples of CRC histopathological images, including (A) debris, (B) mucosa, (C) stroma, (D) adipose tissue, (E) muscle, and (F) lymph nodes.

### 2.3. Performance Metrics

The key evaluation metrics used to validate the proposed models are accuracy, which measures overall correctness, precision, sensitivity, and specificity, which assess the models’ handling of false positives and negatives. The *F*1 score balances precision and recall, and MCC offers a robust metric for imbalanced data. Misclassification rate reflects overall errors, and Cohen’s kappa measures agreement beyond chance. The following equations define each metric used in the evaluation.

(1) $$Accuracy=\frac{TP+TN}{TP+TN+FP+FN}$$

(2) $$Sensitivity\;\left(Recall\right)=\frac{TP}{TP+FN}$$

(3) $$Specificity=\frac{TN}{FP+TN}$$

(4) $$Precision=\frac{TP}{TP+FP}$$

(5) $$F1\;Score=\frac{2\times (Precision\times Sensitivity)}{Precision+Sensitivity}$$

(6) $$MCC=\frac{TP\times TN-FP\times FN}{\surd (\left(TP+FP\right)\times \left(TP+FN\right)\times \left(TN+FP\right)\times \left(TN+FN\right))}$$

(7) $$Misclassification\;Rate=\frac{Number\;of\;misclassified\;samples}{Total\;number\;of\;samples}\times 100$$

(8) $$Kappa=\frac{P_{o\;}-P_{e}}{1-P_{o}}$$

TP  stands for True Positive, TN for True Negative, FP for False Positive, and FN for False Negative. $P_{o\;}$ represents the relatively observed agreement among raters (accuracy). $P_{e}$ represents the expected accuracy.

### 2.4. Model Interpretability

The transparency of understanding the prediction outcomes is essential in medical image analysis to build confidence among medical experts in the classification models. We use the Gradient-Weighted Class Activation Mapping (Grad-CAM) [51], to elucidate model behavior and enhance interpretability. Grad-CAM is a class-preferential localization technique, facilitating the generation of graphical representations of model predictions. By leveraging gradient information passing through the final convolutional layer, Grad-CAM gives values to individual neurons [52], culminating in generating a heatmap. This heatmap is produced through a weighted combination of feature maps, followed by ReLU activation [52]. Grad-CAM creates a rough localization map by using gradients from an output variable, such as the classification score derived from the features in the last convolutional layer [53].

## 3. Results

This section presents experimental setup, evaluation metrics, and results, offering an overview of the methodology and its effectiveness in CRC classification.

### 3.1. Experimental Setup

The experiments were performed on a laptop (13th Gen Intel^®^ Core ™ i9-13900HX, 2200 MHz, 24 Core(s); GPU: NVIDIA GeForce RTX 4060; and RAM of 32 GB) running on Windows 11 Pro, ×64. Python 3.10.13 is utilized with the frameworks of TensorFlow (https://github.com/tensorflow, accessed on 16 June 2025) and Keras (https://keras.io/, accessed on 16 June 2025).

### 3.2. Performance Comparison of Baseline Versus ADFMs

We selected InceptionV3, Xception, and MobileNet for their proven performance and low computational cost in CRC histopathology, excluding models like AlexNet, VGGNet, and ResNet due to limited gains relative to their complexity. To evaluate the performance of the ADFMs, we compared their classification performance with three well-known transfer learning architectures: InceptionV3, Xception, and MobileNet. Each model was fine-tuned on six CRC datasets, and baseline models were tested using pre-trained weights without further fine-tuning. All models were trained with the Adam optimizer and a learning rate of 1 × 10^−4^. Table 3 presents the test accuracy of the baseline and the ADFMs for each dataset. The ADFMs consistently outperformed the individual models across all datasets. Statistical analysis with a 95% confidence interval showed *p*-values > 0.05 after the Shapiro–Wilk normality test. A paired sample *t*-test revealed significant improvement with *p* = 0.009, confirming that ADFMs outperformed the baseline across CRC histopathological datasets.

### 3.3. Proposed Model Performance Metrics by Class Across Six Datasets

**Figure 3:**
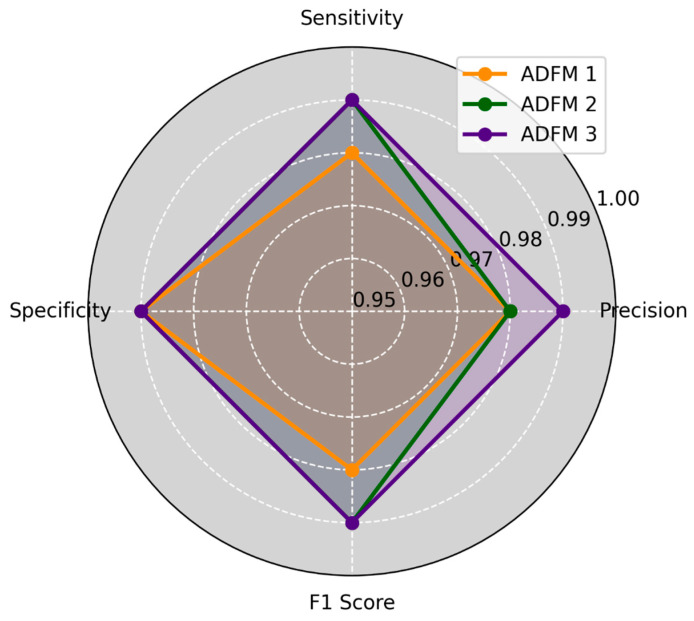
Three ADFMs were employed on three datasets, 1, 3, and 5, with three classes (mucosa, debris, stroma), across four key metrics: precision, sensitivity, specificity, and *F*1-score. ADFM3 shows the highest overall performance. ADFM2 follows closely, performing well but a bit behind ADFM3. ADFM1 indicates slightly lower performance compared to the others.

**Figure 4:**
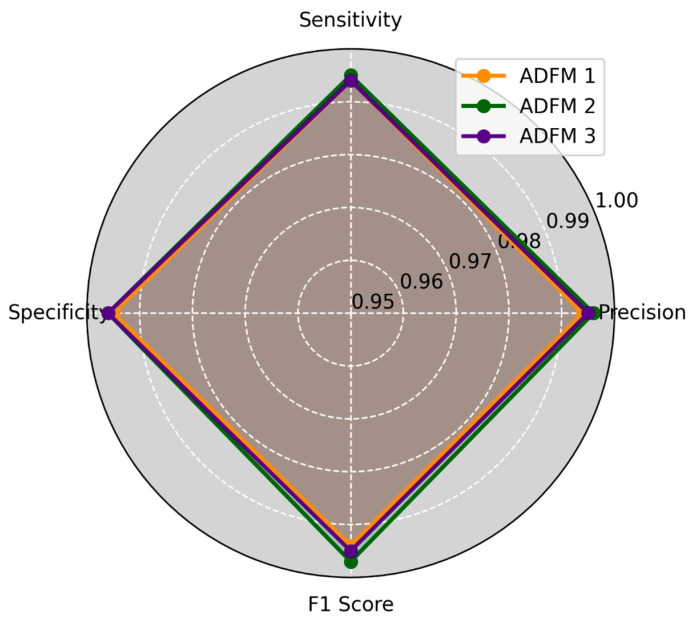
This study applied three ADFMs on three datasets, 2, 4, and 6, with three classes (adipose, muscle, and lymph), across four key metrics: precision, sensitivity, specificity, and *F*1-score. ADFM2 shows slightly higher performance than ADFM1 and ADFM3, and they all perform well compared to each other.

ADFMs were evaluated across six datasets, focusing on the performance of individual classes. Each dataset assessed the models’ ability to categorize histopathological images into specific diagnostic categories, including mucosa, debris, stroma, adipose, muscle, and lymph. The evaluation results are presented in Figure 3 and Figure 4, illustrating the average of how each model performed across different datasets and class types. The metrics reveal the ADFMs’ generalization ability across different data distributions. This analysis identifies performance, enabling a more robust interpretation of each model’s diagnostic potential.

### 3.4. Experimental Results

The CRC histology datasets were sourced from the Royal Hospital in the Sultanate of Oman and a publicly available dataset [27]. The combined samples yield 17,531 histopathological images, all extracted from hematoxylin and eosin-stained whole slides measuring 224 × 224 pixels. The success of this study relied on the CNN models by identifying key hyperparameters and maintaining consistent configurations throughout the model training process, as outlined in Table 4. By standardizing these settings across datasets, this study minimized variability and ensured fair evaluations, thus strengthening the reliability of the findings and enabling an assessment of the models’ performance.

Fusion models: ADFM1, ADFM2, and ADFM3 were selected through extensive experimentation to optimize CRC histopathological image classification. Given the resource-intensive nature of CNN training, accurately estimating time and memory requirements is crucial for efficient model development. To facilitate this, Table 5 and Table 6 present heatmaps comparing the average performance of the models across six datasets.

To assess the practicality of deploying the proposed models in actual clinical environments, we evaluated and benchmarked their computational performance across six datasets on a machine equipped with an NVIDIA GeForce RTX 4060 and 32 GB of RAM. Table 7 summarizes the average inference times, GPU memory consumption, and RAM usage for each model. ADFM1, the largest with (46.9M parameters), shows the highest single image inference time (0.163 s) and batch time (4.3 s). This aligns with expectations that larger models require more computation. In contrast, ADFM2 and 3 have roughly half the parameters (27–28M) and, accordingly, demonstrate faster single image inference times (0.116 and 0.140 s, respectively) and batch times (2.8 and 3.7 s). The average inference time per image in the batch is also the lowest for ADFM2 (0.0064 s), indicating efficient processing. For GPU and RAM usage, GPU memory consumption decreases with model size, with ADFM1 using (889 MB), and ADFM2 and 3 using around (695–740 MB), and RAM consumption remains within a reasonable range. ADFM2 provides the best trade-off between model size, speed, and memory usage. However, when considering the trade-offs, the choice between models should strike a balance between accuracy and memory constraints. Overall, all three ADFMs operate within feasible hardware limits, supporting their potential for clinical integration.

**Figure 5:**
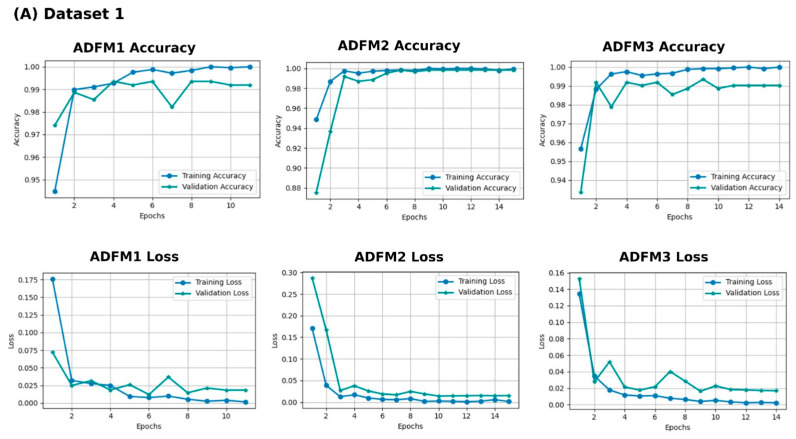

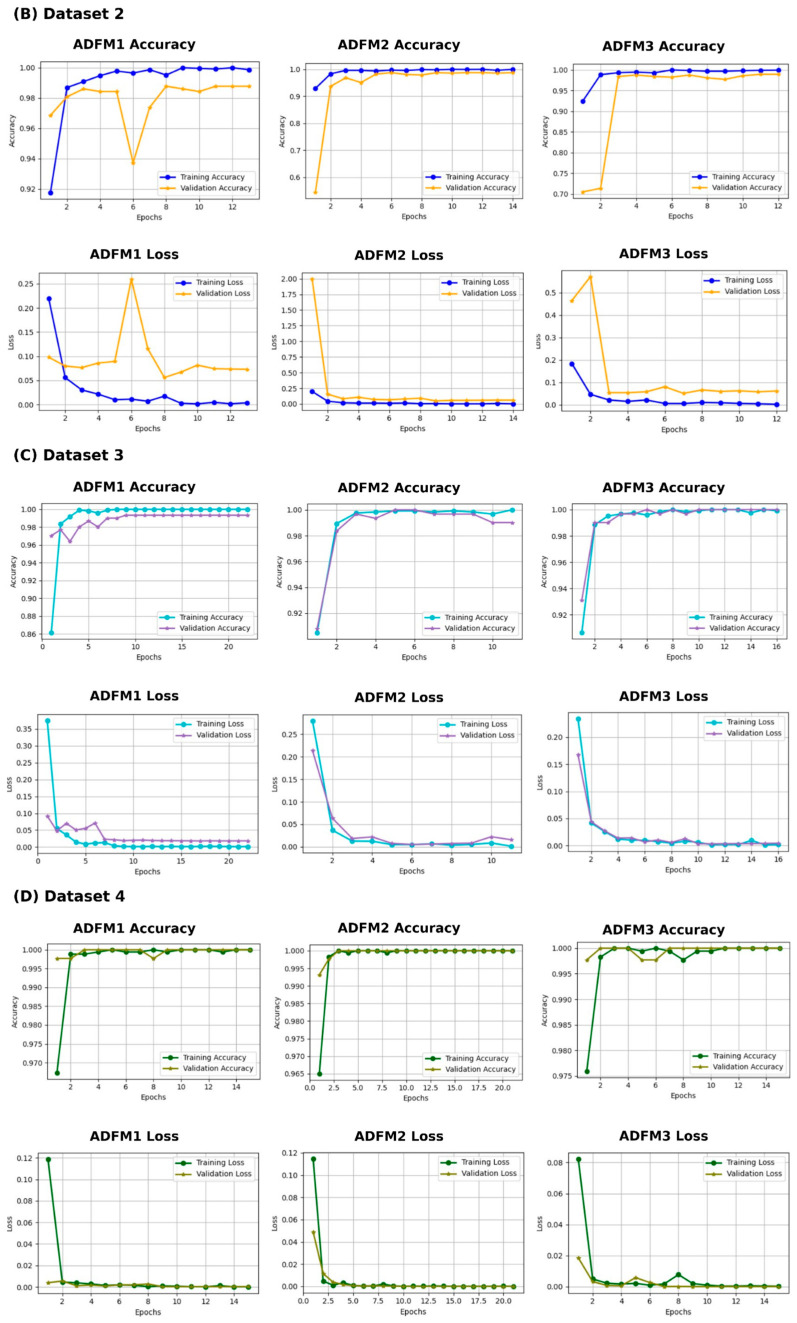

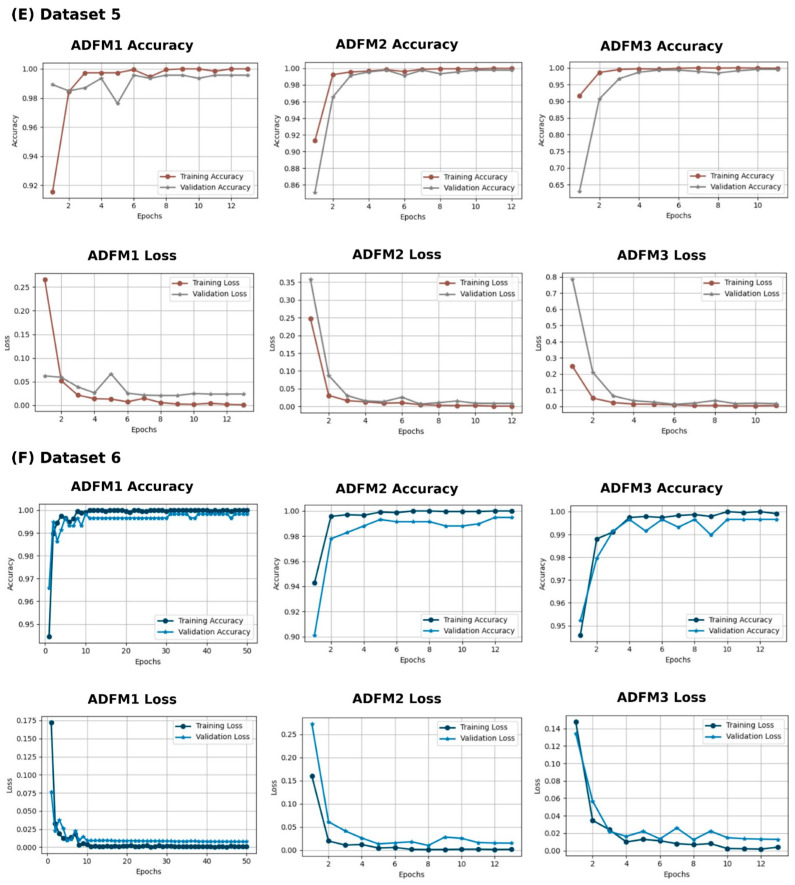
Training and validation performance with three proposed models (ADFM1, ADFM2, and ADFM3) across six datasets (**A**–**F**).

In Figure 5, the training, validation loss, and accuracy curves for the ADFMs across Datasets 1 through 6 are illustrated. These curves indicate that most models exhibit consistent convergence, with no significant gaps between training and validation metrics. However, ADFM1 and ADFM3 show slight variation in performance for (A) Dataset 1, highlighting the need to enhance intra-class variability to better capture subtle differences between similar tissue types. ADFM1 demonstrates more fluctuations in (B) Dataset 2. Additionally, ADFM1 required more epochs to converge in (F) Dataset 6. These observations provide insights into each model’s convergence behavior, highlighting their performance trends and capacity to generalize across diverse datasets.

### 3.5. K-Fold Cross-Validation for ADFMs

Five-fold cross-validation provided a robust evaluation, averaging precision scores across folds for each ADFM on each CRC dataset, resulting in an average precision of 99.6%. The Shapiro–Wilk test (*p* = 0.425) confirmed that the data followed a normal distribution, justifying the use of ANOVA. The test identified a significant difference among datasets (*p* = 0.019), with post hoc comparisons revealing significant variation only between Dataset 2 and Dataset 4 (*p* = 0.008). These findings indicate that the ADFMs performed consistently across datasets, with performance differences attributed to dataset features rather than model variations, proving the proposed models’ robustness and generalizability.

To enhance the statistical robustness of our evaluation, we report additional descriptive metrics for both accuracy and *F*1-score across all ADFMs and datasets. Based on 18 evaluations (three ADFMs across six datasets), the average accuracy and *F*1-score were both 99.6%, with standard deviations of 0.296 and 0.294, respectively. The 95% confidence intervals for the mean accuracy and *F*1-score were [99.5%, 99.8%] and [99.5%, 99.7%], respectively. Furthermore, Table 8, Table 9 and Table 10 present the mean confusion matrix averaged over 5-fold cross-validation. The proposed models demonstrate strong classification performance, with the majority of samples being correctly assigned.

### 3.6. Visualizing Interpretability in CRC Classification Using ADFMs

ADFMs were improved in interpretability using a heatmap-based classification approach. Even though CNNs have advanced image analysis, their predictions often lack transparency. To enhance interpretability and address potential limitations in model transparency, Grad-CAM heatmaps were integrated to visualize the critical regions influencing each model’s decisions. This interpretability component was further reinforced through collaboration with expert pathologists during both data collection and qualitative cross-validation, thereby strengthening the clinical relevance of the approach. The generated heatmaps and superimposed visualizations offered clearer insights into tumor localization across different CRC tissue classes. As illustrated in Table 11, representative examples were assessed by pathologists to evaluate the extent to which the attention maps aligned with diagnostically relevant regions. In the mucosa tissue (A), malignant glands were generally well-recognized; however, the intensity of attention varied across the image. Similarly, in lymph node metastasis (B), the majority of metastatic glands were correctly highlighted, although a few were missed. In contrast, for muscle tissue (C), the attention map effectively recognized smooth muscle fibers but failed to emphasize malignant glands. Finally, in the case of debris (D), the attention map corresponded well with nuclear debris, with only minor omissions at the periphery. These findings highlight the potential of our approach to focus on clinically meaningful regions while also identifying areas where further improvement is needed in challenging tissue types. Table 11 illustrates the heatmaps on CRC images, enabling the identification of the focused region.

## 4. Discussion

The proposed ADFMs offer an advanced CRC histopathological image classification approach by combining deep learning architectures with decision fusion and spatial attention mechanisms. By leveraging three pre-trained networks: Inception V3, Xception, and MobileNet, the models effectively extract features suited for CRC diagnosis. This strategy enables high classification performance across six diverse datasets. 

Table 12 compares the literature on automated methodologies for classifying CRC histopathological images. By benchmarking the ADFMs against the reported techniques, evidence suggests that the ADFMs outperform previous approaches in accuracy and generalizability. The test accuracy rates for ADFM1, ADFM2, and ADFM3 are exceptionally high, averaging 99.25%, 99.4%, and 99.47%, respectively. Also, misclassification across datasets was minimal, with Dataset 1 (545 test samples) showing seven misclassified samples for ADFM1 and four for both ADFM2 and ADFM3. Similarly, in Dataset 2 (506 test samples), ADFM1, ADFM2, and ADFM3 misclassified three, four, and three samples, respectively. For Dataset 3 (270 test samples), ADFM1, ADFM2, and ADFM3 misclassified two, three, and two samples, respectively. In Dataset 4 (385 test samples), none of the models misclassified any samples. For Dataset 5, ADFM1 misclassified 5 out of 408 test samples, while ADFM2 and ADFM3 misclassified 3 and 2 samples. In Dataset 6, with 518 samples, ADFM1, ADFM2, and ADFM3 misclassified 3, 1, and 3 samples, respectively. As discussed with pathologists, we will continue to refine the models through iterative testing and incorporate additional tissue types into each class to improve generalization and accuracy, mitigating the risk of misclassification.

The Grad-CAM visualization method enhances the models’ interpretability, enabling pathologists to validate the models’ focus areas and trust the results. The findings from these models underscore their potential to improve CRC diagnosis, especially in post-surgery scenarios where accurate classification is essential for guiding treatment decisions. The performance of the ADFM and baseline of three individual transfer learning architectures, InceptionV3, Xception, and MobileNet, were compared. The ADFM yielded significant results with a *p*-value of 0.009, providing an automated and consistent approach that has the potential to reduce the subjectivity and variability inherent in manual analysis. However, the fusion architecture used, while demonstrating high performance, introduces computational complexity, which could present challenges in resource-limited environments. This complexity reflects the broader trend in deep neural networks, where exponential growth in model size and the number of parameters significantly elevates memory requirements for storing weights and intermediate outcomes, such as activations and gradients [54,55]. Research directives should validate the models on broader, more diverse clinical datasets to improve generalizability across healthcare settings. Although deep neural networks typically require high computational power during training, their prediction time is generally much faster. However, optimizing computational complexity remains crucial to enable real-time deployment in environments with limited hardware resources. Additionally, integrating multi-modal data and developing more intuitive interpretability techniques will ensure clinicians fully trust and utilize these models for accurate diagnoses in actual scenarios. These advancements could strengthen the robustness and clinical relevance of the models, contributing to enhanced patient outcomes and more accurate CRC diagnoses.

## Figures and Tables

**Table 1:** Pseudocode for attention-based decision fusion. This pseudocode outlines the core logic of the proposed fusion mechanism, where outputs from two pre-trained models are combined using learned attention weights to generate a final classification. The process ensures the adaptive weighting of model contributions for more robust predictions.

Concatenate predictions and feature maps from both models. Compute attention weights using a dense layer with softmax activation. Scale each model’s prediction by its corresponding attention weight. Sum the weighted predictions to produce the final classification. Return the final prediction.

**Table 2:** Detailed descriptions of CRC histopathological image datasets. These datasets reflect a range of tissue types, providing a comprehensive basis for training and evaluating the proposed models.

Class/Dataset	Dataset 1	Dataset 3	Dataset 5
Mucosa	1480	1035	1200
Debris	1030	339	678
Stroma	1119	421	842
**Class/Dataset**	**Dataset 2**	**Dataset 4**	**Dataset 6**
Adipose	1014	1338	1000
Muscle	1239	592	1184
Lymph	1118	634	1268

**Table 3:** Test accuracy comparison of baseline versus ADFMs. This table presents the test accuracy results across all evaluated datasets, comparing baseline architectures with the proposed attention-based decision fusion models. The performance gains highlight the effectiveness of the fusion strategy in enhancing classification accuracy, particularly in the analysis of complex CRC histopathology images.

Dataset	InceptionV3 (%)	Xception (%)	MobileNet (%)	Baseline Avg (%)	ADFM Avg (%)
Dataset 1	97.06	97.61	98.89	97.85	99.08
Dataset 2	97.82	97.23	98.41	97.82	99.33
Dataset 3	96.29	93.70	97.77	95.92	99.13
Dataset 4	100.0	99.74	99.48	99.74	100.00
Dataset 5	96.07	95.83	97.54	96.48	99.18
Dataset 6	97.49	98.06	97.49	97.68	99.55

**Table 4:** ADFM hyperparameters and training configuration. This table summarizes the key hyperparameter settings used across ADFMs. These settings were selected based on empirical tuning to ensure the best performance across the CRC classification.

Hyperparameter	Value
Input image shape	(224, 224, 3)
Initial learning rate	10^−4^
Number of epochs	50
Batch size	32
Dropout rate	0.5
Optimizer	Adam
Loss function	Categorical Cross-Entropy
Fine-tuning	Enabled (Top layers unfrozen)
Number of top layers	20 (Top layers unfrozen for fine-tuning)
Learning rate scheduler	Epoch < 10: No change; Epoch ≥ 10: The learning rate is reduced by a factor of 10 every ten epochs, with a minimum value set to 1 × 10^−6^
Early stopping	Monitor validation loss, patience: 10 epochs, restore best weights: yes

**Table 5:** Three ADFMs were evaluated across ten performance metrics, showing the average performance of each ADFM in three datasets (Dataset 1, Dataset 3, Dataset 5) with three classes (mucosa, debris, stroma).

Metrics (AVG)	ADFM1	ADFM2	ADFM3
Validation Accuracy	99.36	99.54	99.53
Test Accuracy	98.91	99.13	99.33
Validation Loss	0.019	0.012	0.011
Test Loss	0.024	0.027	0.018
MCC	98.29	98.61	98.95
Kappa	98.27	98.64	98.98
Miss-classified Samples	4.66	3.33	2.66
Miss Classification Rate	1.08	0.86	0.65
Early Stopping (Epoch)	15.33	12.66	13.66
Duration per Epoch (Seconds)	21.66	11.83	10

**Table 6:** This table presents three ADFMs evaluated across ten performance metrics, showing the average performance of each ADFM in three datasets (Dataset 2, Dataset 4, and Dataset 6) with three classes (adipose, muscle, and lymph).

Metrics (AVG)	ADFM1	ADFM2	ADFM3
Validation Accuracy	99.53	99.42	99.53
Test Accuracy	99.6	99.66	99.6
Validation Loss	0.0267	0.0253	0.0243
Test Loss	0.0154	0.0073	0.0094
MCC	99.41	99.5	99.41
Kappa	99.41	99.5	99.41
Miss classified Samples	2	1.66	2
Miss classification Rate	0.39	0.32	0.39
Early Stopping (Epoch)	26	16	13.33
Duration per Epoch (Seconds)	16.11	19.83	20.16

**Table 7:** Performance benchmarking results of the proposed ADFMs, which were evaluated across six different datasets using an NVIDIA GPU. Single image, batch inference time, GPU, and RAM usage were recorded during inference. This table summarizes the average performance across all datasets.

Model	Total Params (M)	Single Image Time (s)	Batch Time (s)	Avg Inference Time per Image (s)	GPU Memory Usage (MB)	RAM Usage (MB)
ADFM1	46,879,070	0.1626	4.2952	0.0099	888.81	10,079.91
ADFM2	27,253,502	0.1156	2.7973	0.0064	740.19	10,925.62
ADFM3	28,194,806	0.1398	3.6582	0.0083	695.17	10,944.34

**Table 8:** Mean confusion matrix for ADFM1 model across 5-folds on datasets (1 to 6). Values represent the average per-class predictions, showing ADFM1′s ability to distinguish among CRC histopathological tissue types.

Dataset 1				Dataset 2			
**Actual\Predicted**	Debris	Mucosa	Stroma	**Actual\Predicted**	Adipose	Lymph	Muscle
Debris	205	0.8	0.2	Adipose	202.0	0.4	0.4
Mucosa	0	295.2	0.8	Lymph	0.2	220.4	3.0
Stroma	0	0.8	223	Muscle	0.2	2.6	245.0
**Dataset 3**				**Dataset 4**			
**Actual\Predicted**	Debris	Mucosa	Stroma	**Actual\Predicted**	Adipose	Lymph	Muscle
Debris	67.4	0.2	0.2	Adipose	267.6	0.0	0.0
Mucosa	0.2	206.2	0.6	Lymph	0.0	126.8	0.0
Stroma	0.0	0.6	83.6	Muscle	0.0	0.0	118.4
**Dataset 5**				**Dataset 6**			
**Actual\Predicted**	Debris	Mucosa	Stroma	**Actual\Predicted**	Adipose	Lymph	Muscle
Debris	134.8	0.8	0.0	Adipose	199.8	0.2	0.0
Mucosa	0.2	238.6	1.2	Lymph	0.0	253.2	0.4
Stroma	0.0	0.2	168.2	Muscle	0.2	0.6	236.0

**Table 9:** Mean confusion matrix for ADFM2 model across 5-folds on six datasets, displays averaged predictions per class reflecting ADFM2′s classification behavior across varying CRC tissue distributions.

Dataset 1				Dataset 2			
**Actual\Predicted**	Debris	Mucosa	Stroma	**Actual\Predicted**	Adipose	Lymph	Muscle
Debris	205.4	0.2	0.4	Adipose	202.0	0.2	0.6
Mucosa	0.4	295.0	0.6	Lymph	0.0	221.8	1.8
Stroma	0.8	2.0	221.0	Muscle	0.2	0.6	247.0
**Dataset 3**				**Dataset 4**			
**Actual\Predicted**	Debris	Mucosa	Stroma	**Actual\Predicted**	Adipose	Lymph	Muscle
Debris	67.8	0.0	0.0	Adipose	267.6	0.0	0.0
Mucosa	0.0	206.8	0.2	Lymph	0.0	126.8	0.0
Stroma	0.4	0.4	83.4	Muscle	0.0	0.0	118.4
**Dataset 5**				**Dataset 6**			
**Actual\Predicted**	Debris	Mucosa	Stroma	**Actual\Predicted**	Adipose	Lymph	Muscle
Debris	135.0	0.6	0.0	Adipose	199.4	0.2	0.4
Mucosa	0.2	239.2	0.6	Lymph	0.0	253.6	0.0
Stroma	0.4	0.6	167.4	Muscle	0.0	0.2	236.6

**Table 10:** The mean confusion matrix for the ADFM3 model across 5-folds on the datasets summarizing the average class-wise predictions.

Dataset 1				Dataset 2			
**Actual\Predicted**	Debris	Mucosa	Stroma	**Actual\Predicted**	Adipose	Lymph	Muscle
Debris	205.6	0.0	0.4	Adipose	201.8	0.0	1.0
Mucosa	0.2	295.2	0.6	Lymph	0.4	220.6	2.6
Stroma	0.4	1.4	222.0	Muscle	0.6	1.4	245.8
**Dataset 3**				**Dataset 4**			
**Actual\Predicted**	Debris	Mucosa	Stroma	**Actual\Predicted**	Adipose	Lymph	Muscle
Debris	67.8	0.0	0.0	Adipose	267.6	0.0	0.0
Mucosa	0.0	206.8	0.2	Lymph	0.0	126.8	0.0
Stroma	0.0	0.2	84.0	Muscle	0.0	0.0	118.4
**Dataset 5**				**Dataset 6**			
**Actual\Predicted**	Debris	Mucosa	Stroma	**Actual\Predicted**	Adipose	Lymph	Muscle
Debris	135.0	0.4	0.2	Adipose	199.8	0.0	0.2
Mucosa	0.2	238.8	1.0	Lymph	0.2	252.0	1.4
Stroma	0.2	2.0	166.2	Muscle	0.2	1.0	235.6

**Table 11:** Heatmap visualization of CRC classification: This table presents examples of the tissue class, original image (left), the corresponding feature heatmap (middle), and the superimposed image highlighting areas of focus (right). The heatmaps emphasize the tumor regions, associated features, and the models’ ability to capture relevant characteristics for accurate CRC classification.

Tissue Class	Original Image	Heatmaps	Superimposed Image
(A) Mucosa	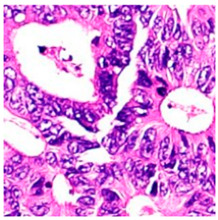	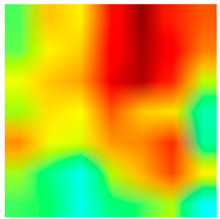	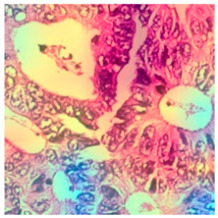
(B) Lymph Node	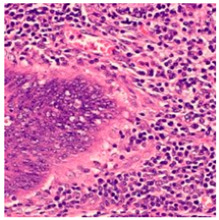	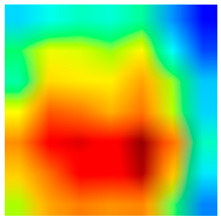	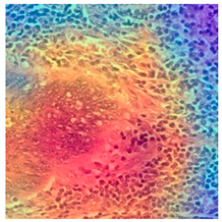
(C) Muscle	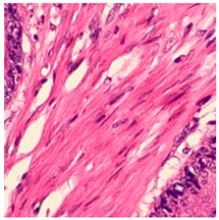	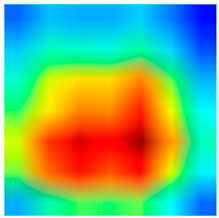	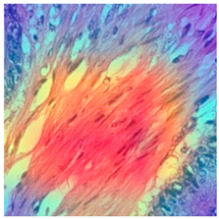
(D) Debris	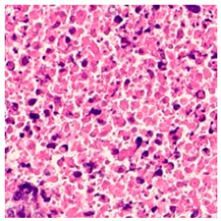	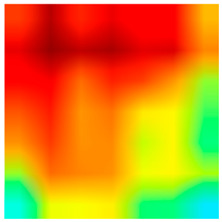	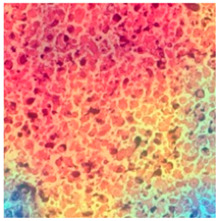

**Table 12:** Overview of automated methods for histopathological image classification. This table summarizes key deep learning-based approaches, facilitating the comparison of the proposed models with existing techniques.

Reference	Method	Dataset	Architecture	Accuracy (%)
[24]	Attention mechanism with Transfer Learning	CRC—Private	VGG16	87.3
[30]	Transfer Learning	CRC— Public	VGG16, ResNet50, adaptive ResNet152	96.16 97.08 98.38
[31]	Transfer Learning	Colon— Public	ResNet50 ResNet18	88 85
[32]	Custom CNN	Colon— Public	CNN	99.75
[33]	Custom CNN	Lung and Colon— Public	CNN	96.33
[34]	Custom CNN	Lung and Colon—Public	CNN	56–99.50
[35]	Transfer Learning	CRC—Private	Inception V3	95.1
[36]	Transfer Learning	CRC—Public	ResNet50	94.86
[37]	Transfer Learning	CRC—Public	VGG19	91.2
[38]	Transfer Learning	CRC—Public	ResNet50	94.8
[39]	Transfer Learning	Colon—Private	VGG19, DenseNet201, EfficientNetB7	94.17
Proposed Models	Spatial attention mechanism and decision fusion with Transfer Learning	CRC—Private CRC—Public	ADFM1 ADFM2 ADFM3	98.71–100.00 98.88–100.00 99.25–100.00

## Data Availability

The dataset used in this study is publicly available at Zenodo: https://zenodo.org/records/1214456 (accessed on 16 June 2025). The private dataset and the code supporting the findings are available from the corresponding author upon reasonable request.

## Abbreviations

The following abbreviations are used in this manuscript:

ILSVRC	ImageNet Large Scale Visual Recognition Competition
CNN	Convolutional Neural Network
CRC	Colorectal Cancer
Grade-CAM	Gradient-Weighted Class Activation Mapping
ADFM	Attention Decision Fusion Models

## References

[1] LeCun Y., Bengio Y., Hinton G. (2015). Deep Learning. Nature.

[2] Krizhevsky A., Sutskever I., Hinton G.E. (2017). ImageNet Classification with Deep Convolutional Neural Networks. Commun. ACM.

[3] Lecun Y., Bottou L., Bengio Y., Haffner P. (1998). Gradient-Based Learning Applied to Document Recognition. Proc. IEEE.

[4] Simonyan K., Zisserman A. (2014). Very Deep Convolutional Networks for Large-Scale Image Recognition. arXiv.

[5] Szegedy C., Liu W., Jia Y., Sermanet P., Reed S., Anguelov D., Erhan D., Vanhoucke V., Rabinovich A. Going Deeper with Convolutions. Proceedings of the 2015 IEEE Conference on Computer Vision and Pattern Recognition (CVPR).

[6] Chollet F. Xception: Deep Learning with Depthwise Separable Convolutions. Proceedings of the 2017 IEEE Conference on Computer Vision and Pattern Recognition (CVPR).

[7] He K., Zhang X., Ren S., Sun J. Deep Residual Learning for Image Recognition. Proceedings of the 2016 IEEE Conference on Computer Vision and Pattern Recognition (CVPR).

[8] Howard A.G., Zhu M., Chen B., Kalenichenko D., Wang W., Weyand T., Andreetto M., Adam H. (2017). MobileNets: Efficient Convolutional Neural Networks for Mobile Vision Applications. arXiv.

[9] Arora R., Sharma S., Kumar B. (2022). Colorectal Cancer: Risk Factors and Potential of Dietary Probiotics in Its Prevention. Proc. Indian Natl. Sci. Acad..

[10] Chodoff A., Smith K.C., Shukla A., Blackford A.L., Ahuja N., Johnston F.M., Peairs K.S., Ngaiza J.R., Warczynski T., Nettles B. (2022). Colorectal Cancer Survivorship Care Plans: Variations in Documentation and Posttreatment Surveillance Recommendations. J. Surg. Oncol..

[11] Xi Y., Xu P. (2021). Global Colorectal Cancer Burden in 2020 and Projections to 2040. Transl. Oncol..

[12] Pataki B.Á., Olar A., Ribli D., Pesti A., Kontsek E., Gyöngyösi B., Bilecz Á., Kovács T., Kovács K.A., Kramer Z. (2022). HunCRC: Annotated Pathological Slides to Enhance Deep Learning Applications in Colorectal Cancer Screening. Sci. Data.

[13] Hossain M.S., Karuniawati H., Jairoun A.A., Urbi Z., Ooi D.J., John A., Lim Y.C., Kibria K.M.K., Mohiuddin A.K.M., Ming L.C. (2022). Colorectal Cancer: A Review of Carcinogenesis, Global Epidemiology, Current Challenges, Risk Factors, Preventive and Treatment Strategies. Cancers.

[14] Chen H., Li C., Li X., Rahaman M.M., Hu W., Li Y., Liu W., Sun C., Sun H., Huang X. (2022). IL-MCAM: An Interactive Learning and Multi-Channel Attention Mechanism-Based Weakly Supervised Colorectal Histopathology Image Classification Approach. Comput. Biol. Med..

[15] Ai S., Li C., Li X., Jiang T., Grzegorzek M., Sun C., Rahaman M.M., Zhang J., Yao Y., Li H. (2021). A State-of-the-Art Review for Gastric Histopathology Image Analysis Approaches and Future Development. BioMed Res. Int..

[16] De Matos J., Ataky S., De Souza Britto A., Soares De Oliveira L., Lameiras Koerich A. (2021). Machine Learning Methods for Histopathological Image Analysis: A Review. Electronics.

[17] Jansen-Winkeln B., Barberio M., Chalopin C., Schierle K., Diana M., Köhler H., Gockel I., Maktabi M. (2021). Feedforward Artificial Neural Network-Based Colorectal Cancer Detection Using Hyperspectral Imaging: A Step towards Automatic Optical Biopsy. Cancers.

[18] Kumar N., Gupta R., Gupta S. (2020). Whole Slide Imaging (WSI) in Pathology: Current Perspectives and Future Directions. J. Digit. Imaging.

[19] Pacal I., Karaboga D., Basturk A., Akay B., Nalbantoglu U. (2020). A Comprehensive Review of Deep Learning in Colon Cancer. Comput. Biol. Med..

[20] Cai L., Gao J., Zhao D. (2020). A Review of the Application of Deep Learning in Medical Image Classification and Segmentation. Ann. Transl. Med..

[21] Atasever S., Azginoglu N., Terzi D.S., Terzi R. (2023). A Comprehensive Survey of Deep Learning Research on Medical Image Analysis with Focus on Transfer Learning. Clin. Imaging.

[22] Goncalves T., Rio-Torto I., Teixeira L.F., Cardoso J.S. (2022). A Survey on Attention Mechanisms for Medical Applications: Are We Moving Toward Better Algorithms?. IEEE Access.

[23] Pan S.J., Yang Q. (2010). A Survey on Transfer Learning. IEEE Trans. Knowl. Data Eng..

[24] Zhou P., Cao Y., Li M., Ma Y., Chen C., Gan X., Wu J., Lv X., Chen C. (2022). HCCANet: Histopathological Image Grading of Colorectal Cancer Using CNN Based on Multichannel Fusion Attention Mechanism. Sci. Rep..

[25] Sung H., Ferlay J., Siegel R.L., Laversanne M., Soerjomataram I., Jemal A., Bray F. (2021). Global Cancer Statistics 2020: GLOBOCAN Estimates of Incidence and Mortality Worldwide for 36 Cancers in 185 Countries. CA Cancer J. Clin..

[26] Dif N., Elberrichi Z. (2020). A New Deep Learning Model Selection Method for Colorectal Cancer Classification. Int. J. Swarm Intell. Res..

[27] Kather J.N., Krisam J., Charoentong P., Luedde T., Herpel E., Weis C.-A., Gaiser T., Marx A., Valous N.A., Ferber D. (2019). Predicting Survival from Colorectal Cancer Histology Slides Using Deep Learning: A Retrospective Multicenter Study. PLoS Med..

[28] Zhou S.K., Greenspan H., Davatzikos C., Duncan J.S., Van Ginneken B., Madabhushi A., Prince J.L., Rueckert D., Summers R.M. (2021). A Review of Deep Learning in Medical Imaging: Imaging Traits, Technology Trends, Case Studies With Progress Highlights, and Future Promises. Proc. IEEE.

[29] Vrbancic G., Podgorelec V. (2020). Transfer Learning with Adaptive Fine-Tuning. IEEE Access.

[30] Naga Raju M.S., Rao B.S. (2022). Colorectal Multi-Class Image Classification Using Deep Learning Models. Bull. EEI.

[31] Sarwinda D., Paradisa R.H., Bustamam A., Anggia P. (2021). Deep Learning in Image Classification Using Residual Network (ResNet) Variants for Detection of Colorectal Cancer. Procedia Comput. Sci..

[32] Yildirim M., Cinar A. (2022). Classification with Respect to Colon Adenocarcinoma and Colon Benign Tissue of Colon Histopathological Images with a New CNN Model: MA_ColonNET. Int. J. Imaging Syst. Technol..

[33] Masud M., Sikder N., Nahid A.-A., Bairagi A.K., AlZain M.A. (2021). A Machine Learning Approach to Diagnosing Lung and Colon Cancer Using a Deep Learning-Based Classification Framework. Sensors.

[34] Sakr A.S., Soliman N.F., Al-Gaashani M.S., Pławiak P., Ateya A.A., Hammad M. (2022). An Efficient Deep Learning Approach for Colon Cancer Detection. Appl. Sci..

[35] Xu L., Walker B., Liang P.-I., Tong Y., Xu C., Su Y.C., Karsan A. (2020). Colorectal Cancer Detection Based on Deep Learning. J. Pathol. Inform..

[36] Tsai M.-J., Tao Y.-H. (2021). Deep Learning Techniques for the Classification of Colorectal Cancer Tissue. Electronics.

[37] Vidyun A.S., Srinivasa Rao B., Harikiran J., Bhateja V., Satapathy S.C., Travieso-González C.M., Aradhya V.N.M. (2021). Histopathological Image Classification Using Deep Neural Networks with Fine-Tuning. Data Engineering and Intelligent Computing.

[38] Sun K., Chen Y., Bai B., Gao Y., Xiao J., Yu G. (2023). Automatic Classification of Histopathology Images across Multiple Cancers Based on Heterogeneous Transfer Learning. Diagnostics.

[39] Karabulut İ., Selen R., Yağanoğlu M., Özmen S. (2024). Recognition of Colon Polyps (Tubular Adenoma, Villous Adenoma) and Normal Colon Epithelium Histomorphology with Transfer Learning. Eurasian J. Med..

[40] Ilhan H.O., Serbes G., Aydin N. (2022). Decision and Feature Level Fusion of Deep Features Extracted from Public COVID-19 Data-Sets. Appl. Intell..

[41] Ali I., Muzammil M., Haq I.U., Amir M., Abdullah S. (2021). Deep Feature Selection and Decision Level Fusion for Lungs Nodule Classification. IEEE Access.

[42] Khayyat M.M., Elrefaei L.A. (2020). Manuscripts Image Retrieval Using Deep Learning Incorporating a Variety of Fusion Levels. IEEE Access.

[43] El-Ghandour M., Obayya M., Yousif B. (2024). Breast Cancer Histopathology Image Classification Using an Ensemble of Optimized Pretrained Models with a Trainable Ensemble Strategy Classifier. Res. Biomed. Eng..

[44] Deng J., Dong W., Socher R., Li L.-J., Li K., Fei-Fei L. ImageNet: A Large-Scale Hierarchical Image Database. Proceedings of the 2009 IEEE Conference on Computer Vision and Pattern Recognition.

[45] Kavitha M.S., Gangadaran P., Jackson A., Venmathi Maran B.A., Kurita T., Ahn B.-C. (2022). Deep Neural Network Models for Colon Cancer Screening. Cancers.

[46] Kandel I., Castelli M. (2020). How Deeply to Fine-Tune a Convolutional Neural Network: A Case Study Using a Histopathology Dataset. Appl. Sci..

[47] Li J., Wang P., Zhou Y., Liang H., Lu Y., Luan K. (2021). A Novel Classification Method of Lymph Node Metastasis in Colorectal Cancer. Bioengineered.

[48] Ohata E.F., Chagas J.V.S.D., Bezerra G.M., Hassan M.M., De Albuquerque V.H.C., Filho P.P.R. (2021). A Novel Transfer Learning Approach for the Classification of Histological Images of Colorectal Cancer. J. Supercomput..

[49] Szegedy C., Vanhoucke V., Ioffe S., Shlens J., Wojna Z. Rethinking the Inception Architecture for Computer Vision. Proceedings of the 2016 IEEE Conference on Computer Vision and Pattern Recognition (CVPR).

[50] Bankhead P., Loughrey M.B., Fernández J.A., Dombrowski Y., McArt D.G., Dunne P.D., McQuaid S., Gray R.T., Murray L.J., Coleman H.G. (2017). QuPath: Open Source Software for Digital Pathology Image Analysis. Sci. Rep..

[51] Selvaraju R.R., Cogswell M., Das A., Vedantam R., Parikh D., Batra D. Grad-CAM: Visual Explanations from Deep Networks via Gradient-Based Localization. Proceedings of the 2017 IEEE International Conference on Computer Vision (ICCV).

[52] Shovon M.S.H., Islam M.J., Nabil M.N.A.K., Molla M.M., Jony A.I., Mridha M.F. (2022). Strategies for Enhancing the Multi-Stage Classification Performances of HER2 Breast Cancer from Hematoxylin and Eosin Images. Diagnostics.

[53] Dabass M., Vashisth S., Vig R. (2022). A Convolution Neural Network with Multi-Level Convolutional and Attention Learning for Classification of Cancer Grades and Tissue Structures in Colon Histopathological Images. Comput. Biol. Med..

[54] Sevilla J., Heim L., Ho A., Besiroglu T., Hobbhahn M., Villalobos P. Compute Trends Across Three Eras of Machine Learning. Proceedings of the 2022 IEEE International Joint Conference on Neural Networks (IJCNN).

[55] Steiner B., Elhoushi M., Kahn J., Hegarty J. MODeL: Memory Optimizations for Deep Learning. Proceedings of the Proceedings of the 40th International Conference on Machine Learning, PMLR.

